# A machine learning model for robust prediction of sepsis-induced coagulopathy in critically ill patients with sepsis

**DOI:** 10.3389/fcimb.2025.1579558

**Published:** 2025-06-06

**Authors:** Jia Sun, Lixin Zhang, Zhaotang Gong, Hongling Ma, Dan Wu, Rihan Wu, Guleng Siri

**Affiliations:** ^1^ Department of Pharmacy, Inner Mongolia People’s Hospital, Hohhot, Inner Mongolia Autonomous Region, China; ^2^ Department of Pharmacy, Baotou Medical College, Baotou, Inner Mongolia Autonomous Region, China; ^3^ Real-World Research Center, Inner Mongolia Academy of Medical Sciences, Hohhot, Inner Mongolia Autonomous Region, China

**Keywords:** sepsis, sepsis-induced coagulopathy, risk factor, machine learning, predict

## Abstract

**Introduction:**

Sepsis-induced coagulopathy (SIC) is a common disease in patients with sepsis. It denotes higher mortality rates and a poorer prognosis in these patients. This study aimed to develop a practical machine learning (ML) model for the prediction of the risk of SIC in critically ill patients with sepsis.

**Methods:**

In this retrospective cohort study, patients were extracted from the Medical Information Mart for Intensive Care IV (MIMIC-IV) database and the Inner Mongolia Autonomous Region People’s Hospital database. Sepsis and SIC were defined based on the Sepsis-3 criteria and the criteria developed based on the International Society of Thrombosis and Haemostasis (ISTH), respectively. We compared nine ML models using the Sequential Organ Failure Assessment (SOFA) score in terms of SIC prediction ability. Optimal model selection was based on the superior performance metrics exhibited by the model on the training dataset, the internal validation dataset, and the external validation dataset.

**Results:**

Of the 15,479 patients in MIMIC-IV included in the final cohort, a total of 6,036 (38.9%) patients developed SIC during sepsis. We selected 17 features to construct ML prediction models. The gradient boosting machine (GBM) model was deemed optimal as it achieved high predictive accuracy and reliability across the training, internal, and external validation datasets. The areas under the curve of the GBM model were 0.773 (95%CI = 0.765–0.782) in the training dataset, 0.730 (95%CI = 0.715–0.745) in the internal validation dataset, and 0.966 (95%CI = 0.938–0.994) in the external validation dataset. The Shapley Additive Explanations (SHAP) values illustrated the prediction results, indicating that total bilirubin, red cell distribution width (RDW), systolic blood pressure (SBP), heparin, and blood urea nitrogen (BUN) were risk factors for progression to SIC in patients with sepsis.

**Conclusions:**

We developed an optimal and operable ML model that was able to predict the risk of SIC in septic patients better than the SOFA scoring models.

## 1 Introduction

Sepsis is a serious syndrome that accompanies severe infection and has significant morbidity and mortality in the intensive care unit (ICU), often initiated by a localized infection that induces a systemic inflammatory response syndrome (SIRS) (Levi et al., 2003). It is characterized by a life-threatening organ dysfunction, which is caused by a dysregulation of the host’s response to infection. It is estimated that more than 19 million people suffer from sepsis each year, and it has become one of the major threats to human mortality (Mithal et al., 2022). Currently, the hospital mortality of adults with sepsis is approximately 189/100,000 person-years, while the ICU mortality is as high as over 42% (Fleischmann-Struzek et al., 2020).

Sepsis-induced coagulopathy (SIC) is regarded as the earliest and the most common complication of sepsis, which leads to the formation of thrombus and coagulation dysfunction (Qin and Dun, 2021). Coagulopathy is present in 50%–70% of patients with sepsis (MaChado and Silva, 2006). It is a significant complication of sepsis that increases the risk of thrombosis, exacerbates organ failure, and raises the mortality rates (Levi and Ten Cate, 1999; Levi and van der Poll, 2017; Lyons et al., 2018). SIC mortality reaches 23.1% (Iba et al., 2019b). These studies underscore the importance of promptly identifying risk factors that lead to SIC.

SIC criteria were developed by members of the Scientific and Standardization Committee (SSC) on disseminated intravascular coagulation (DIC) of the International Society of Thrombosis and Haemostasis (ISTH) in 2017 (Iba et al., 2017) (**Supplementary Table S1**), which were designed to identify patients with “sepsis and coagulation disorders.” The criteria are a scoring system. SIC is defined as a score ≥4. It was observed that the mortality rate increased along with the increase in the SIC score, and it surpassed 30% when the score reached 4 (Iba et al., 2017). SIC is more relevant than DIC for the updated Sepsis-3 criteria (Iba et al., 2019a). It is therefore of utmost importance to identify patients with SIC early. However, the SIC score mainly serves as a diagnostic system, and there is still a lack of reliable predictive tools for SIC in clinical practice.

In recent years, the prevalence of electronic health records (EHRs) and the development of artificial intelligence (AI) have provided opportunities for clinical medical research (Ngiam and Khor, 2019), and the medical domain has witnessed significant advancements in AI technology, with machine learning (ML) algorithms assuming a pivotal role as a fundamental component of AI. ML models are algorithms that enable computers to learn from data and make predictions. They are categorized into supervised, unsupervised, semi-supervised, and reinforcement learning. Supervised models use labeled data for training, while unsupervised models find patterns in unlabeled data. Semi-supervised models combine both, while reinforcement models learn through trial and error. These models are used in various fields such as healthcare, finance, and marketing to solve complex problems and make data-driven decisions (LeCun et al., 2015). At present, scholars are primarily dedicated to investigating the development and verification of the mortality and prognostic models for critically ill patients with SIC. However, there is a lack of research on a clinical risk prediction model for critically ill patients with SIC.

To bridge this knowledge gap, this study was conducted to investigate the risk factors that influence coagulopathy in patients with sepsis. More specifically, nine ML algorithms and Sequential Organ Failure Assessment (SOFA) scoring were employed to develop and validate early warning models for critically ill patients with SIC, subsequently evaluating their predictive efficacy to identify the most optimal model.

This study utilized both domestic and international medical big data analysis to identify the risk factors for patients with SIC and to establish an operable prediction model. The aim was to identify the early risk factors for the development of SIC, thereby enabling timely and effective interventions and preventive measures to hinder the progression of sepsis to SIC, ultimately improving patient survival rates.

## 2 Methods

### 2.1 Ethical approval

The Medical Information Mart for Intensive Care IV (MIMIC-IV) database is a third-party, anonymized publicly available database with preexisting Institutional Review Board (IRB) approval. For the Inner Mongolia Autonomous Region People’s Hospital data, approval was obtained from the Ethics Committee of the same hospital.

### 2.2 Source of data and participants

An open and free critical care database, the MIMIC-IV, which contained comprehensive clinical data of patients admitted to the Beth Israel Deaconess Medical Center in Boston, Massachusetts, between 2008 and 2019, was retrieved (Goldberger et al., 2000). The other database comprises de-identified health data from ICUs across the Inner Mongolia Autonomous Region between 2015 and 2020. The study was reported according to the recommendations of the Transparent Reporting of a Multivariable Prediction Model for Individual Prognosis or Diagnosis (TRIPOD) statement (Collins et al., 2015).

For sepsis, we retrieved adult sepsis patients (≥18 years old) as defined according to the Sepsis-3 criteria: 1) existing evidence of suspected or confirmed infection and 2) SOFA score ≥2. The exclusion criteria were: 1) age <18 years; 2) pregnant women; 3) patients with congenital coagulopathy; 4) the coagulation function was frequently affected by the pathological state of the tumors and the chemotherapeutic agent used (thus, patients with various cancer types were excluded); and 5) patients who died or were discharged within 24 h after ICU admission.

For SIC, on the basis of all eligible patients with sepsis, SIC patients were defined as those fulfilling the ISTH criteria. Patients were considered to display SIC when they had a total SIC score ≥4 with a total score of the prothrombin time/international normalized ratio (PT-INR) and platelet count parameters >2 during the sepsis (Iba et al., 2017).

### 2.3 Data extraction and processing

Data extraction was performed using PostgreSQL (version 4.21) and STATA (version 18.1) software. The following data were extracted from the MIMIC-IV database and the Inner Mongolia Autonomous Region People’s Hospital database: 1) demographic data; 2) first care unit; 3) outcomes; 4) severity score, including the SOFA and SIC scores; 5) the mean values of vital signs and the poorest laboratory test value during the first 24 h after ICU admission; and 6) infectious sites defined using Navicat Premium 16 software. For the prediction of SIC, the MIMIC-IV database collected 50 variables (**Supplementary Table S2**) and the Inner Mongolia Autonomous Region People’s Hospital database collected 59 variables, including the patient characteristics (age and gender), vital signs (heart rate, respiratory rate, mean arterial pressure, and SpO2), and laboratory data [PT, albumin, anion gap, blood urea nitrogen (BUN), red blood cell count, and glucose]. Comorbidities were also collected based on the recorded International Classification of Diseases, Ninth Revision (ICD-9), combined with the Tenth Revision (ICD-10) diagnosis codes (hypertension, diabetes mellitus, chronic obstructive pulmonary disease, coronary heart disease, and liver disease). Lastly, data on medications such as heparin and continuous renal replacement therapy (CRRT), as well as mechanical ventilation (MV), were collected.

The feature engineering was completed in three steps (Zhou et al., 2024). Firstly, missing value identification and processing. In this study, the “VIM” package was used to recognize the distribution of missing values. Moreover, features with more than 20% missing values were removed, such as albumin, C-reactive protein (CRP), and d-dimer. For the remaining features, missing values were imputed using the “mice” package in R. Secondly, outlier identification and processing. All outliers were not processed as preserving outliers in medical data is critical to building accurate prediction models and meeting clinical needs. Outliers might reflect a true pathological state, and their removal could cause the model to lose key risk factors, reducing its predictive power. In addition, outliers might contain samples of high predictive value, and their exclusion weakens the ability of the model to identify rare but high-risk cases. The retention of outliers ensures the integrity and validity of the dataset, making the model better adapted to the diversity of actual clinical scenarios. At the same time, the retention of outliers helps the model to stay close to clinical needs, which aids physicians in identifying high-risk patients and in implementing timely interventions. Thirdly, feature selection for model construction. Univariate logistic feature selection was first performed on all the included variables, followed by multivariate logistic feature selection. Variables with a *p*-value <0.05 in the univariate analysis were included in the multivariate analysis. A *p*-value <0.05 in the multivariate analysis was considered statistically significant. This “univariate first, then multivariate” feature selection strategy can reduce the number of features while maintaining model performance, thereby improving the interpretability and efficiency of the model.

### 2.4 Statistical analysis

The main outcome was the diagnosis of SIC during sepsis. Continuous variables are presented as the mean (standard deviation) or median (interquartile range, IQR), depending on the distribution of the data, and were analyzed using Student’s *t*-test or the rank-sum test for continuous variables. The chi-square test or Fisher’s exact test was used for categorical variables, as appropriate.

The MIMIC-IV database was randomly assigned with 70% for training and 30% for internal validation, while the Inner Mongolia People’s Hospital was used for external validation. Nine ML methods [i.e., logistic regression (LR), light gradient boosting machine (LightGBM), eXtreme gradient boosting (XGBoost), support vector machine (SVM), categorical boosting (CatBoost), adaptive boosting (Adaboost), neural network (NN), *k*-nearest neighbors (KNN), and gradient boosting machine (GBM)] and the SOFA severity scoring system were respectively used to develop models for the prediction of risk factors for SIC. The assessment process was performed using 10-fold cross-validation. The area under the receiver operating characteristic (ROC) curve (AUC), the area under the precision–recall curve (AUPRC), and the accuracy, sensitivity, specificity, predictive, and F1 scores were all calculated to evaluate the predictive performance of each model. In addition, calibration plots were drawn using the bootstrap method, and decision curve analysis (DCA) was performed for each ML model in the two databases. The best model was selected through an overall comparison. Subsequently, fine-grained hyperparameter adjustment was performed for the best model using the Bayesian optimization algorithm. This algorithm is an efficient constrained global optimization tool (Wu et al., 2019). The optimized model was defined as the full model. The Shapley Additive Explanations (SHAP) approach is commonly applied to explain the output of the ML model (Liu et al., 2021). External validation of the full models was performed in the Inner Mongolia People’s Hospital. The SOFA scores were assessed to predict the risk of SIC and were compared with the ML models in both the internal and external validations.

**Figure 1:**
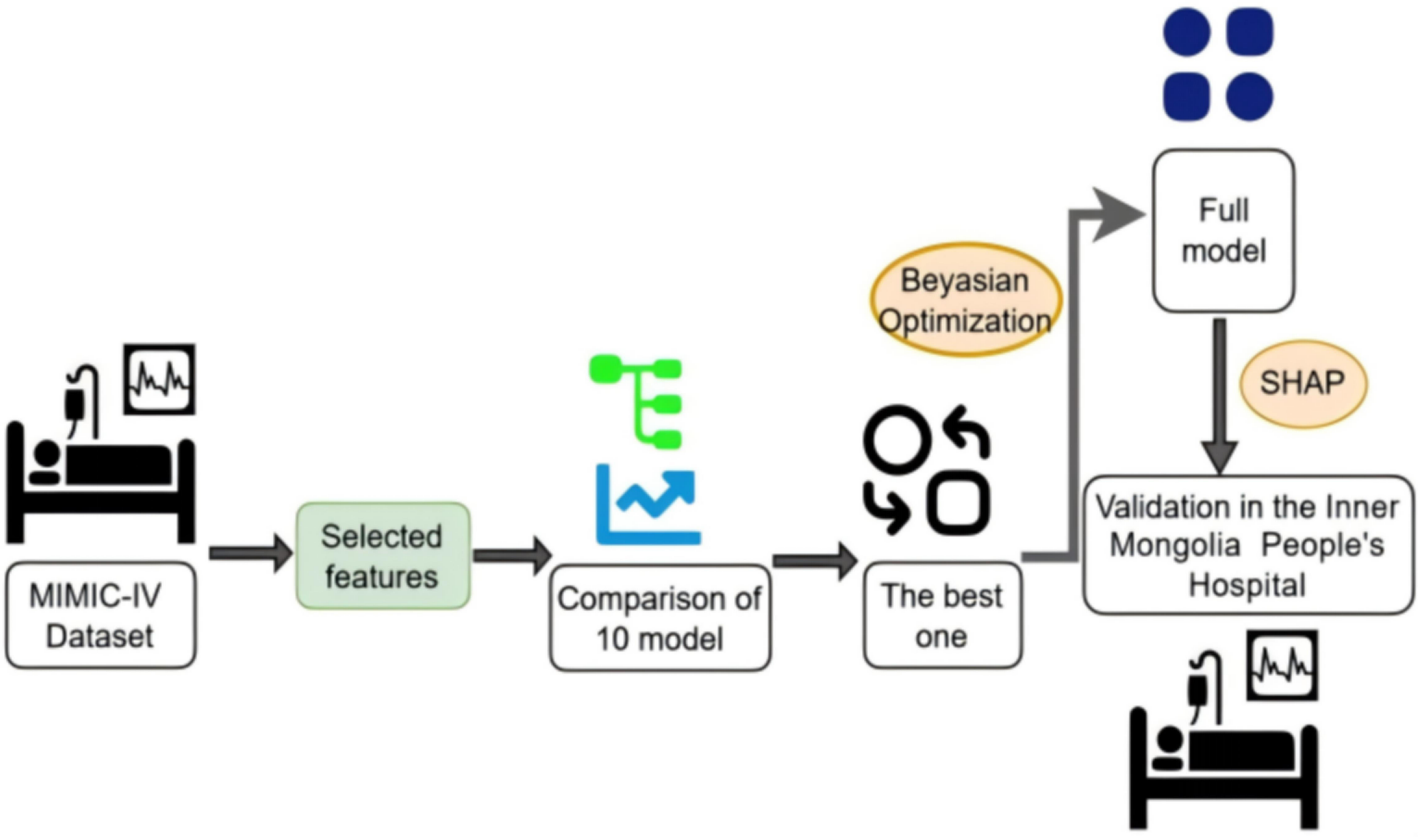
Framework of the prediction model. A total of 17 variables were selected through feature selection in the Medical Information Mart for Intensive Care-IV (MIMIC-IV) database. We compared the discrimination of nine machine learning models using 10-fold cross-validation. The model with the best overall performance was selected. Fine-grained hyperparameter adjustment was performed using Bayesian optimization. The Shapley Additive Explanations (SHAP) values were used to explain the output of the full model. This full model was validated in the Inner Mongolia People’s Hospital.

All statistical analyses were performed using R software (version 4.4.2). The framework of the prediction models is shown in **Figure 1**.

## 3 Results

### 3.1 Baseline characteristics

**Figure 2:**
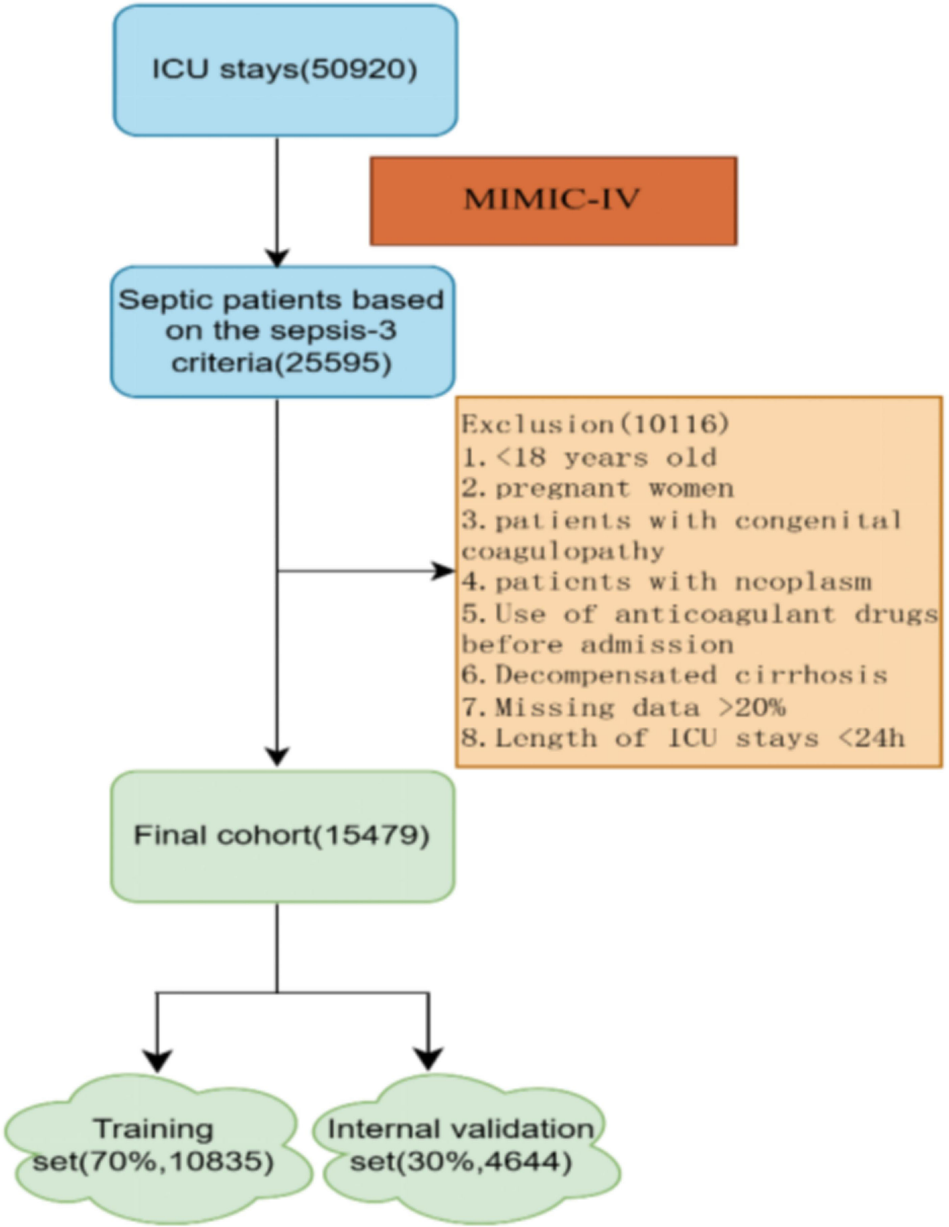
Flowchart of the patient selection.

As shown in **Figure 2**, of the 25,595 patients with sepsis in MIMIC-IV, 15,479 were included in the final cohort, with 10,835 patients in the training set and 4,644 patients in the internal validation set. A total of 6,036 patients developed SIC during sepsis, while 9,443 patients did not. A cohort of 212 patients with sepsis in the Inner Mongolia People’s Hospital was included as an external dataset.

Variable values on the first day of sepsis in MIMIC-IV were analyzed, and differences in the characteristics were compared (**Supplementary Table S2**). The SIC group had a higher rate of comorbidities. Moreover, those in the SIC group were older [71 (59–81) *vs*. 68 (57–79), *p* < 0.001] and had higher Charlson comorbidity index [6 (4–8) *vs*. 5 (3–7), *p* < 0.001], higher creatine [1.3 (0.9–2.1) *vs*. 1 (0.8–1.5), *p* < 0.001], longer PT [17.25 (15.2–22.6) *vs*. 12.6 (11.8–13.55), *p* < 0.001] and activated partial thromboplastin time (PTT) [34.4 (29.7–44.2) *vs*. 28.8 (26.3–32.6), *p* < 0.001], lower platelets (PLT) [151 (87–243) *vs*. 202 (152–269), *p* < 0.001], higher heart rate [87.266 (16.889) *vs*. 83.385 (15.288), *p* < 0.001] and respiration rate [19.32 (17.04-22.36) vs. 18.46 (16.52-20.91), p < 0.001], had more CRRT (7.77% *vs*. 2.51%; *p* < 0.001) and use of vasoactive drugs (17.15% *vs*. 7.76%, *p* < 0.001), and showed slightly higher numbers of urinary (15.23% *vs*. 14.04%, *p* < 0.05) and blood infections (0.83% *vs*. 0.43%, *p* < 0.05).

### 3.2 Comparison of the nine models and the SOFA scores

**Figure 3:**
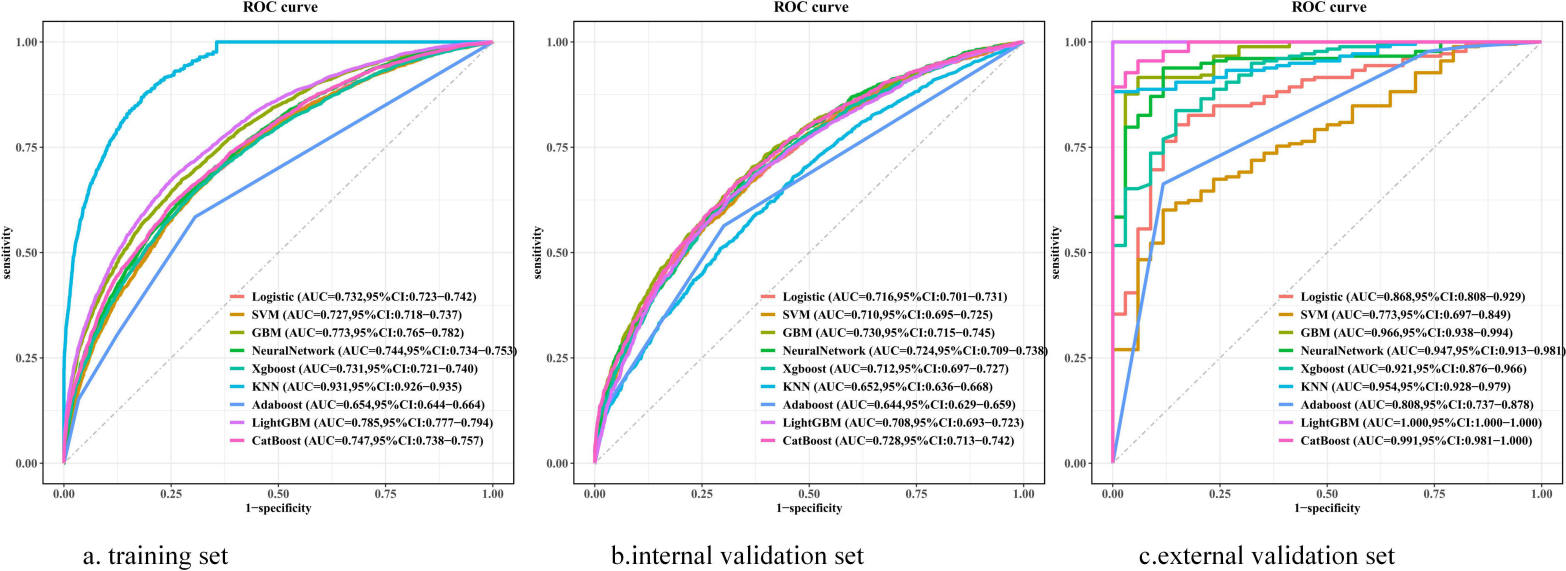
Receiver operating characteristic curves shows the predictive performance of nine machine learning models in predicting the risk factors of SIC. **(A)** Receiver Operating Characteristic curves of various models on the training set. **(B)** Receiver Operating Characteristic curves of various models on the internal validation dataset. **(C)** Receiver Operating Characteristic curves of various models on the external validation dataset. Logistic, logistic regression; SVM, support vector machine; GBM, gradient boosting machine; Neural Network, artificial neural network; XGBoost, eXtreme gradient boosting; KNN, k-nearest neighbors; Adaboost, adaptive boosting; LightGBM, light gradient boosting machine; CatBoost, categorical boosting; AUC, area under the receiver operating characteristic curve; 95%CI, 95% confidence interval.

**Figure 4:**
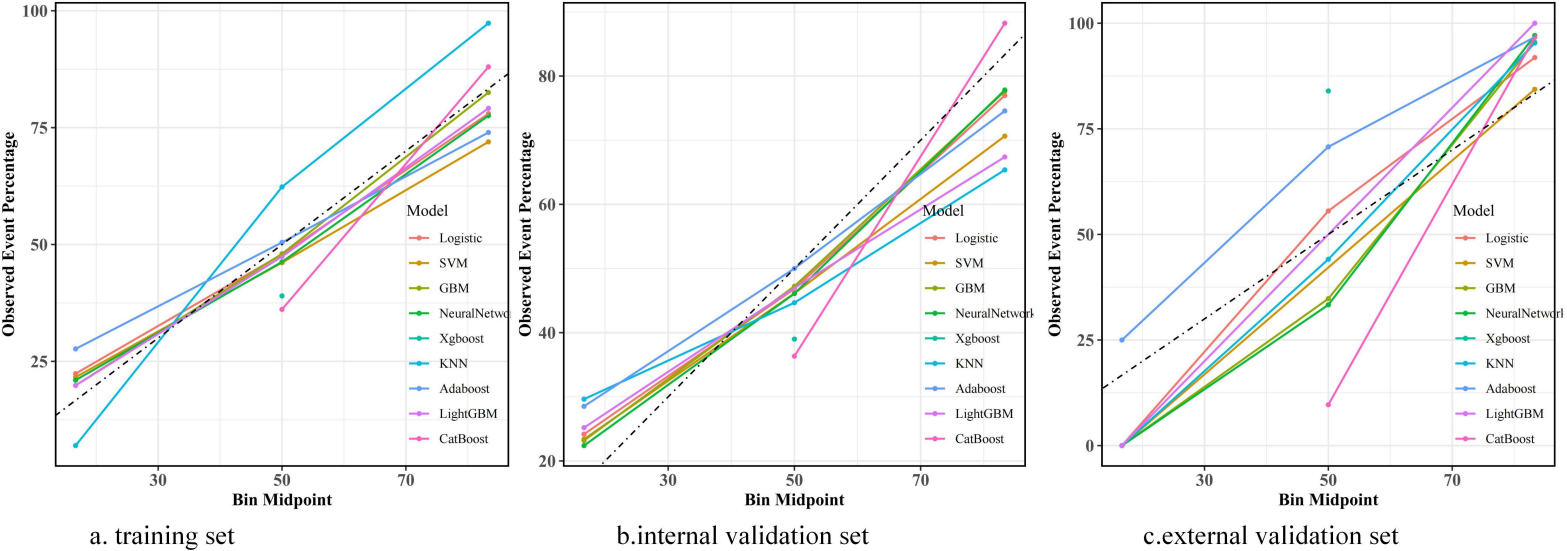
Calibration curves of the nine prediction models across different datasets. **(A)** Performance of the models on the training set. **(B)** Results on the internal validation dataset. **(C)** Assessment outcomes on the external validation dataset. Logistic, logistic regression; SVM, support vector machine; GBM, gradient boosting machine; Neural Network, artificial neural network; XGBoost, eXtreme gradient boosting; KNN, k-nearest neighbors; Adaboost, adaptive boosting; LightGBM, light gradient boosting machine; CatBoost, categorical boosting.

**Figure 5:**
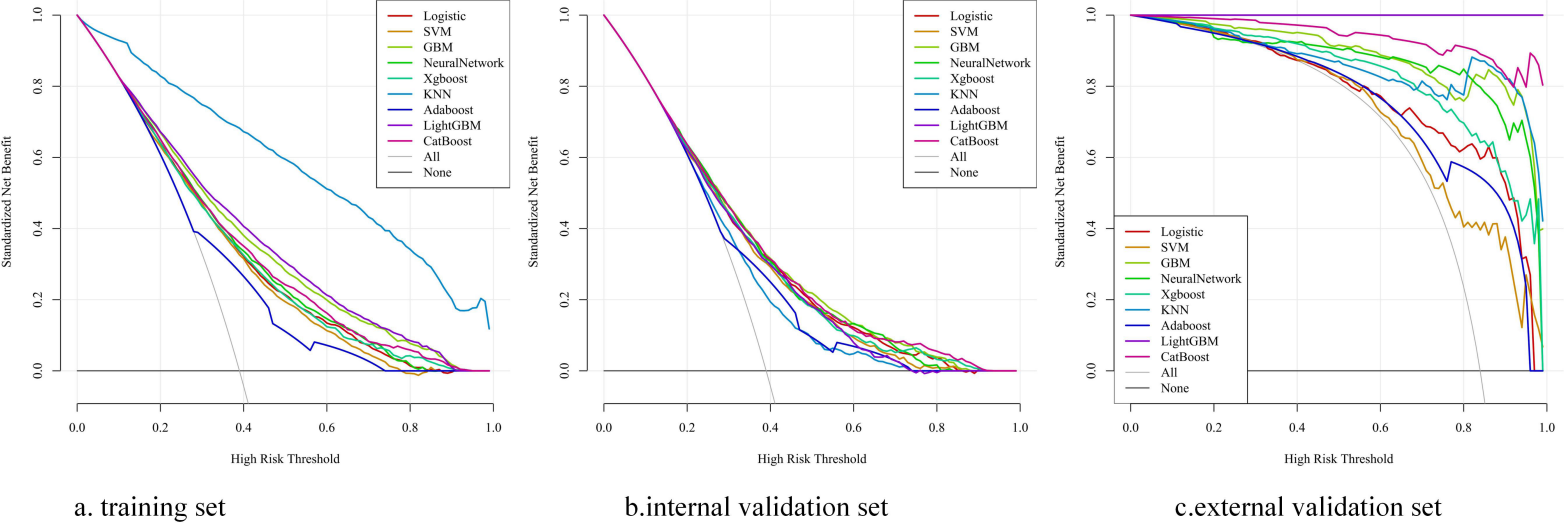
The Decision Curve Analysis (DCA) graph is utilized to compare the clinical utility of various machine learning models in predicting the risk factors for SIC. **(A)** Decision curve analysis of various models on the training set. **(B)** Decision curve analysis of various models on the internal validation dataset. (C) Decision curve analysis of various models on the external validation dataset. Logistic, logistic regression; SVM, support vector machine; GBM, gradient boosted models; Neural Network, artificial neural network; XGBoost, eXtreme gradient boosting; KNN, k-nearest neighbors; Adaboost, adaptive boosting; LightGBM, light gradient boosting machine; CatBoost, categorical boosting.

Before construction of the prediction model, 12 features were initially screened out using univariate logistic regression followed by multivariate logistic regression: heparin, coronary, CRRT, vasoactive drug, PT, anion gap, respiration rate (resp_rate), red cell distribution width (RDW), systolic blood pressure (SBP), mean arterial pressure (mean blood pressure, MBP), mean corpuscular volume (MCV), and total bilirubin (**Supplementary Table S3**). However, PT was deleted from the list of features, while BUN, diabetes, sodium, aspartate aminotransferase (AST), mean corpuscular hemoglobin (MCH), and temperature were included. Thus, 17 features were eventually used to build the model. We utilized nine ML models—LR, XGBoost, SVM, Adaboost, KNN, LightGBM, CatBoost, NN, and GBM—with the 17 features mentioned above to predict the risk factors for SIC in patients with sepsis. The predictive performance of the various models is listed in **Table 1**. The AUCs of the GBM model varied in the training dataset (0.773, 95%CI = 0.765–0.782), the internal validation dataset (0.730, 95%CI = 0.715–0.745), and the external validation dataset (0.966, 95%CI = 0.938–0.994), which showed excellent performance on the overall indicators (**Figures 3a–c**). In addition, the AUCs of the SOFA scores in the different datasets were as follows: 0.504 (95%CI = 0.493–0.504) in the training dataset, 0.511 (95%CI = 0.494–0.511) in the internal validation dataset, and 0.853 (95%CI = 0.791–0.853) in the external validation dataset. The calibration curves are presented in **Figures 4a–c**, while the decision curve analysis (DCA) graphs are shown in **Figures 5a–c**. The bias-corrected lines lightly deviated from the ideal line, indicating good agreement between the prediction and observation. Firstly, the AUC was employed as the primary metric, complemented by secondary metrics such as sensitivity and specificity. Secondly, the DCA framework was utilized to assess the net benefit across various risk thresholds, thereby quantifying the practical utility of the model in clinical decision-making within critical care medicine. Thirdly, a calibration curve was constructed to evaluate the accuracy of the model predictions. Finally, through a comprehensive multidimensional assessment, the GBM algorithm was identified as the optimal model, and subsequent in-depth analyses were conducted.

**Table 1:** Predictive performance of each model in the training, internal validation, and external validation datasets.

Model	AUC	Threshold	Accuracy	Sensitivity	Specificity	Precision	F1
Training set							
Logistic	0.732	0.367	0.673	0.664	0.679	0.569	0.613
SVM	0.727	0.395	0.676	0.636	0.702	0.577	0.605
GBM	0.773	0.387	0.705	0.673	0.725	0.61	0.64
Neural Network	0.744	0.416	0.689	0.613	0.738	0.599	0.606
XGBoost	0.731	0.499	0.674	0.653	0.687	0.572	0.61
KNN	0.931	0.370	0.839	0.868	0.819	0.755	0.807
Adaboost	0.654	0.202	0.651	0.584	0.694	0.55	0.567
LightGBM	0.785	0.383	0.718	0.68	0.743	0.629	0.653
CatBoost	0.747	0.601	0.697	0.613	0.752	0.612	0.612
SOFA	0.496	0.399	0.546	0.402	0.607	0.301	0.344
Internal validation dataset							
Logistic	0.716	0.396	0.678	0.594	0.731	0.585	0.59
SVM	0.710	0.351	0.645	0.693	0.614	0.535	0.603
GBM	0.730	0.359	0.668	0.671	0.667	0.563	0.612
Neural Network	0.724	0.364	0.66	0.683	0.645	0.552	0.61
XGBoost	0.712	0.499	0.671	0.596	0.719	0.576	0.586
KNN	0.652	0.406	0.63	0.509	0.708	0.527	0.518
Adaboost	0.644	0.202	0.646	0.564	0.699	0.545	0.554
LightGBM	0.708	0.359	0.664	0.63	0.686	0.562	0.594
CatBoost	0.728	0.594	0.672	0.649	0.687	0.57	0.607
SOFA	0.489	0.380	0.521	0.392	0.636	0.491	0.436
External validation dataset							
Logistic	0.868	0.787	0.811	0.803	0.853	0.966	0.877
SVM	0.773	0.836	0.646	0.601	0.882	0.964	0.74
GBM	0.966	0.764	0.92	0.916	0.941	0.988	0.95
Neural Network	0.947	0.697	0.929	0.938	0.882	0.977	0.957
XGBoost	0.921	0.503	0.84	0.837	0.853	0.968	0.898
KNN	0.954	0.702	0.901	0.882	1.000	1.000	0.937
Adaboost	0.808	0.822	0.698	0.663	0.882	0.967	0.787
LightGBM	1.000	0.500	1.000	1.000	1.000	1.000	1.000
CatBoost	0.991	0.688	0.934	0.927	0.971	0.994	0.959
SOFA	0.853	0.795	0.802	0.953	0.435	0.803	0.872

Logistic, logistic regression; SVM, support vector machine; GBM, generalized boosted model; Neural Network, artificial neural network; XGBoost, eXtreme gradient boosting; KNN, k-nearest neighbors; Adaboost, adaptive boosting; LightGBM, light gradient boosting machine; CatBoost, categorical boosting; SOFA, Sequential Organ Failure Assessment score; AUC, area under the receiver operating characteristic curve.

### 3.3 Explanation of risk factors

**Figure 6:**
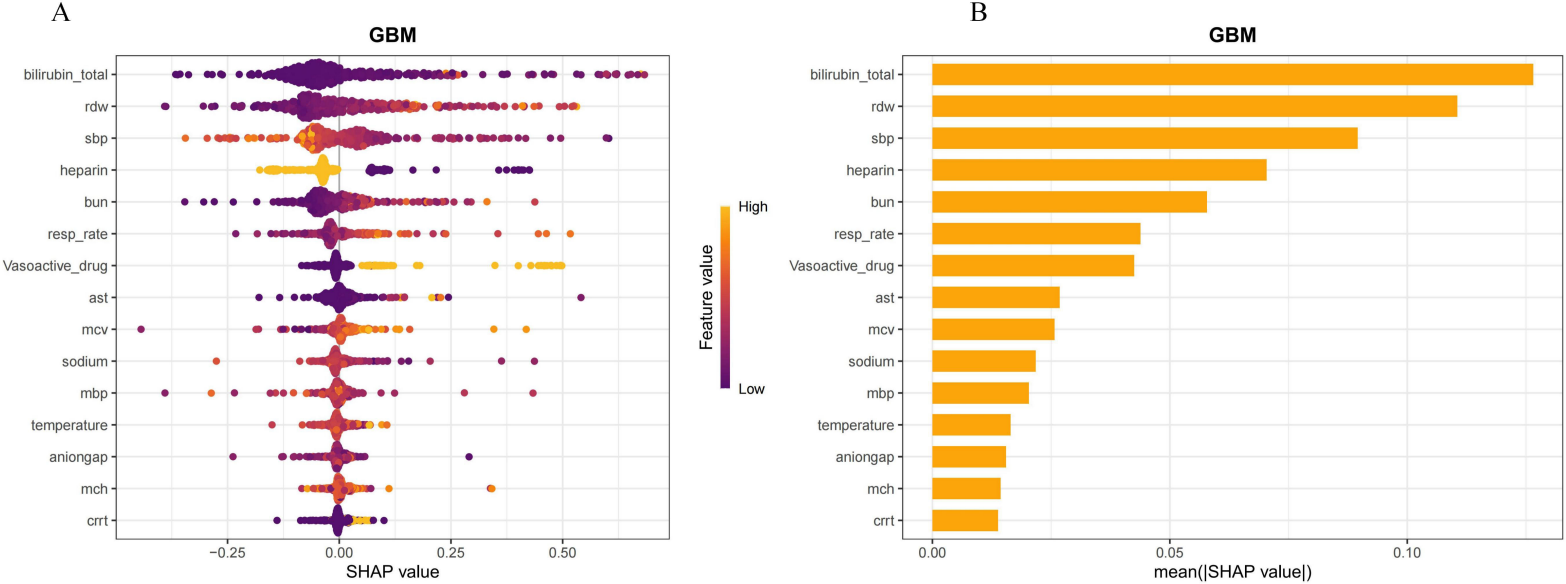
Interpretation of the generalized boosted model (GBM). **(A)** Feature importance ranking based on the Shapley Additive Explanations (SHAP) values. The position on the *y*-axis implies the importance ranking, while the *x*-axis reflects the association between each feature value and the corresponding SHAP value. **(B)** Importance ranking of the included features according to the mean (|SHAP value|). RDW, red blood cell distribution width; SBP, systolic blood pressure; MBP, mean arterial pressure; BUN, blood urea nitrogen; resp_rate, respiration rate; MCH, mean corpuscular hemoglobin; AST, aspartate aminotransferase; CRRT, continuous renal replacement therapy.

The importance scores of the 17 features used in the GBM model were calculated to identify the critical features (**Figure 6A**). The position on the *y*-axis implied the importance ranking, and the *x*-axis reflected the association between each feature value and the corresponding SHAP value (Kishor et al., 2015). For instance, high heparin levels generally have SHAP values below 0, suggesting that heparin use may reduce the risk of SIC in patients with sepsis. In addition, high values of RDW correspond to SHAP values that are mostly greater than 0, suggesting that RDW values may promote the incidence of SIC. **Figure 6B** displays the ranking of the features based on the average absolute SHAP value. The permutation importance results indicated that the top five risk features were total bilirubin, RDW, SBP, heparin, and BUN. Diabetes and coronary disease may demonstrate low SHAP values (i.e., marginal contributions to the prediction outputs) within the model, which failed to meet the predefined display threshold in the visualization.

### 3.4 Interpretation of individual predictions

**Figure 7:**
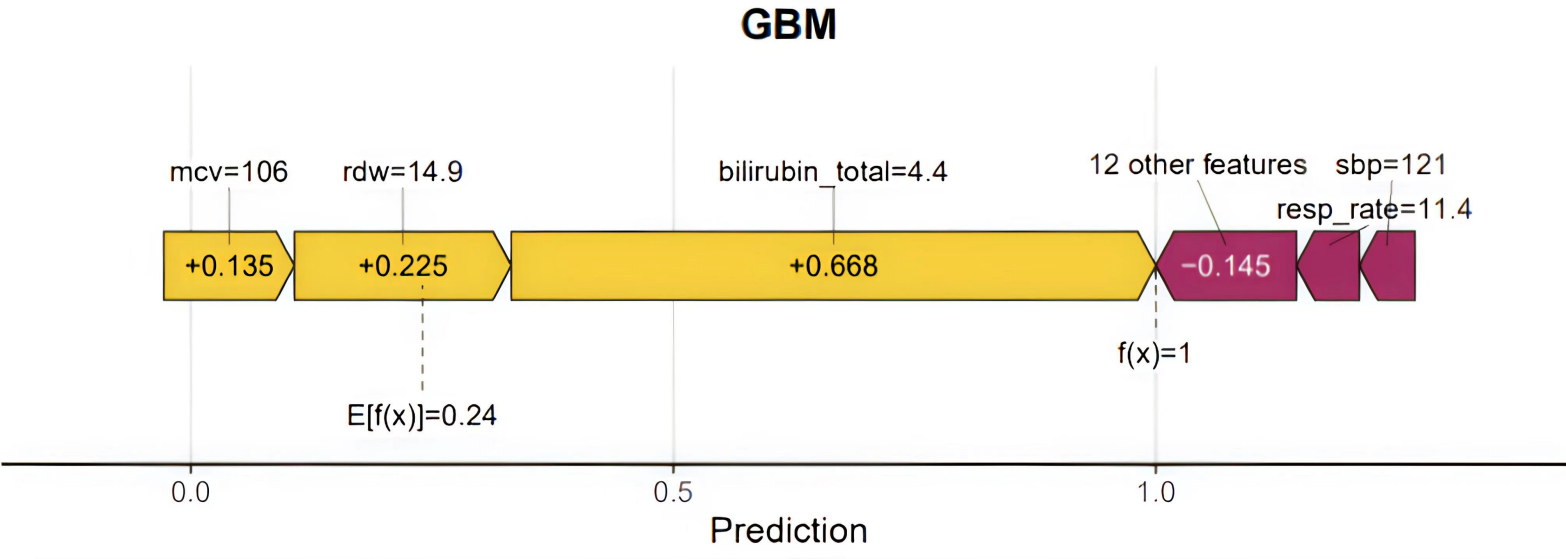
The notation *f*(*x*) = 1 represents the predicted value of the model for a specific instance or sample. *E*[*f*(*x*)] = 0.24 denotes the average predicted value, or the expected value, of the model across the dataset. The *bars in yellow and red* represent the risk factors and the protective factors, respectively; *longer bars* denote greater feature importance. Here, these values are the model outputs before the SoftMax layer and, therefore, are not equal to the final predicted probabilities. This figure shows the explanation for a high-risk instance. RDW, red blood cell distribution width; SBP, systolic blood pressure; MBP, mean arterial pressure; BUN, blood urea nitrogen; resp_rate, respiration rate; MCH, mean corpuscular hemoglobin; AST, aspartate aminotransferase; CRRT, continuous renal replacement therapy.

**Figure 7** shows the SHAP force plot, which was used to explain the individual prediction results of the GBM model. The SHAP value measures the contribution degree of each feature to the prediction results of the model. The yellow bars represent positive SHAP values (increasing the probability of the predicted risk), while the red bars represent negative SHAP values (decreasing the probability of the predicted risk). The length of the bars is proportional to the strength of the contribution. As shown in the figure, *f*(*x*) = 1 represents the final prediction result, which is equal to the baseline value *E*[*f*(*X*)] = 0.24 plus the sum of the SHAP values of all variables. The total bilirubin value was 4.4, the RDW value was 14.9, and the MCV value was 106, which made positive contributions of 0.668, 0.225, and 0.135, respectively, to the prediction. In contrast, the combined effect of the other 12 features was a negative contribution of 0.145, while the SBP value of 121 and the resp_rate value of 11.4 both had relatively small negative contributions.

## 4 Discussion

At present, the medical community is confronted by the absence of dependable predictive tools designed to predict the onset of coagulopathy in patients with sepsis. This gap highlights a critical need for the development of advanced predictive tools that can provide early and accurate detection of coagulation disorders, thereby facilitating timely and effective interventions. The quest for such tools is intensifying, with the goal of decreasing the development of SIC in septic patients by enabling clinicians to anticipate and address the complex challenges associated with septic coagulopathy.

In this study, we developed and validated ML models using 17 selected features—diabetes, coronary, CRRT, heparin, vasoactive drug, temperature, sodium, resp_rate, anion gap-min, BUN-max, total bilirubin, MCV, MCH, RDW, MBP, SBP, and AST—to predict the risk of SIC. The administration of vasoactive drugs could alter the coagulation function through various mechanisms (Mohammadi et al., 2017; Petch et al., 2021). Diabetes mellitus, serum sodium levels, AST, body temperature, MCH, and BUN were included in this study following systematic discussion and multifactorial analysis. Although (Liu et al., 2022) did not directly investigate the relationship between diabetes mellitus and SIC, their findings demonstrated that blood glucose variability exacerbates microthrombosis through oxidative stress and endothelial glycocalyx injury, thereby providing a rationale for its inclusion. (Han et al., 2024) confirmed the association between serum sodium fluctuations and coagulation dysfunction in sepsis, revealing that such fluctuations destabilize coagulation homeostasis by impairing the vascular endothelial glycocalyx integrity, while hypernatremia further aggravates coagulation disorders via platelet activation and coagulation factor stimulation. (Wang et al., 2025) established that elevated AST levels indicate hepatic dysfunction, which directly reduces clotting factor production (given the liver’s synthesis of 80%–90% of coagulation factors) and promotes a prothrombotic state. Body temperature, validated by (Zhou et al., 2024) through ML models, serves not only as a sensitive marker of systemic inflammation but also as a predictive biomarker for sepsis-related coagulation dysfunction. In addition (Wang et al., 2025), identified that MCH influences the erythrocyte oxygen-carrying capacity, indirectly linking it to coagulation abnormalities in the context of sepsis-associated hypoxia and metabolic dysregulation. Both (Wang et al., 2025) and (Zhou et al., 2024) emphasized the pathophysiological significance of BUN, demonstrating that sepsis-induced renal injury (reflected by an elevated BUN) disrupts coagulation regulation. Based on the “multi-organ crosstalk hypothesis” of SIC, the inclusion of BUN is supported by its role in the bidirectional interplay between renal dysfunction and coagulation derangements.

The above features could be easily collected within 24 h after ICU admission. Under the condition of ensuring accuracy, it achieved practicality as far as possible. The GBM model predicted SIC based on these 17 clinical variables, which demonstrated excellent predictive performance in the training, internal validation, and external validation datasets. The predictive performance of the models was assessed using a comprehensive set of metrics including the AUC, accuracy, sensitivity, specificity, positive predictive value (PPV), negative predictive value (NPV), and the F1 score. Our ML model was compared with the SOFA score for the following reasons. Firstly, the SOFA score is a well-established standardized ICU tool that evaluates organ dysfunction and disease severity through physiological indicators. It exhibits significant correlations with SIC and mortality (Iba et al., 2017), thereby serving as a pivotal clinical benchmark. Secondly, its globally recognized consensus and systematic framework make it particularly suitable for the validation of ML models. Finally, by comparing the AUC metrics in SIC risk prediction, it can be determined whether novel models surpass traditional scoring systems while improving the interpretability and clinical applicability. Furthermore, given its proven prognostic value for organ failure (Fleischmann-Struzek et al., 2020), the SOFA score is extensively utilized as a baseline control in studies of sepsis-related complications. Moreover, the SHAP approach was employed to interpret the predictions of the GBM model, offering clinicians insights into the decision-making process of the model and aiding in the timely identification of sepsis patients at high risk of developing SIC for proactive intervention. Currently, ML plays a crucial role in the early warning and prognostic prediction of diseases (Vellido, 2020; Jiang et al., 2021). These algorithms can analyze complex and nonlinear data and even make real-time predictions based on time series, which cannot be completed using traditional regression analysis. GBM is an ensemble learning algorithm that combines multiple weak learners, typically decision trees, to form a robust prediction model. It optimizes the loss function through gradient descent, iteratively adding weak learners that focus on correcting the residuals of the previous model. This process of sequential addition and correction enhances the predictive accuracy of the model, making GBM highly effective for both regression and classification tasks. However, as algorithms advance, the complexity of models grows, which can make them harder to interpret. This complexity is commonly described as creating a “black box” effect, potentially hindering the adoption of ML in the medical and healthcare sectors (Lippi et al., 2009). The challenge lies in balancing the sophistication of ML models with the need for transparency and interpretability, which are crucial for their acceptance and effective use in clinical practice.

By using the SHAP value to interpret the GBM model, which is different from traditional feature importance, its most significant advantage is that it can reflect the permutation of importance and illustrate the positive and negative effects of the included features. As shown in **Figure 5**, it was found to be the most important variable in the prediction of SIC, followed by total bilirubin. Elevated total bilirubin levels may indicate that sepsis has affected the liver, impairing its ability to synthesize clotting factors and leading to coagulation dysfunction in patients (Patel et al., 2015). The second risk factor was the RDW, the value of which reflects the size heterogeneity of the erythrocytes and indicates the body’s response to oxidative stress and inflammation (Zhang et al., 2020). In recent years, a growing number of research studies have shown the potential value of RDW in predicting the prognosis of sepsis (Fu et al., 2018; Ling et al., 2021; Wang and Hsu, 2021). Low SBP leads to hypoperfusion and endothelial damage, which activates the coagulation system and increases the risk of thrombosis, thereby resulting in SIC. Heparin is a potent anticoagulant that enhances the activity of antithrombin (AT), inhibiting the activation of clotting factors and thereby reducing thrombus formation. In patients with sepsis, heparin can effectively prevent and treat coagulopathy (Zou et al., 2022). found that the early administration of prophylactic heparin in patients with sepsis can reduce mortality and improve outcomes. Moreover, anticoagulant therapies in patients without SIC should be avoided due to the increased risk of bleeding with no survival benefit (Yu et al., 2024). In patients with sepsis, an elevated BUN may indicate an impaired renal function, which is part of multi-organ dysfunction syndrome (MODS). Renal insufficiency can further exacerbate coagulopathy, as the kidneys play a crucial role in clearing metabolic waste from the blood and in regulating electrolyte balance (Zhou et al., 2024; Wang et al., 2025). Therefore, it is crucial to monitor and manage the total bilirubin, RDW, SBP, heparin, and BUN, as well as other indicators, in patients with sepsis in clinical practice to prevent the occurrence of SIC.

The SOFA score, the platelet count, and the PT-INR have been found to be significant in numerous studies, making the monitoring of these three indicators essential. Any abnormality in these indicators should raise suspicion for the possibility of SIC. In addition to these three indicators, we also propose that attention should be given to total bilirubin, SBP, heparin use, RDW, and BUN. Abnormalities in these additional indicators should also prompt consideration of the potential for SIC.

## 5 Strengths and limitations

This research has several notable strengths. Firstly, GBM has shown excellent performance in various applications due to its high predictive accuracy, ability to handle complex relationships, support for missing values, automatic feature selection, and flexibility, making it particularly suitable for tasks that require high interpretability. Secondly, the SHAP solves the “black box” problem well for ML models. Thirdly, based on the SHAP values, we ranked the risk factors and illustrated the positive and negative risk factors that lead to SIC. Moreover, this study is the first to incorporate a domestic sepsis database combined with the MIMIC database to explore the risk factors for SIC. However, several limitations of this study should be considered. Firstly, only septic adults in ICUs were included. Secondly, the ROC curve of the GBM model is not the largest; however, the GBM model was chosen for the prediction of the risk factors that lead to SIC based on a comprehensive consideration; thus, further clinical experience and medical judgment should be recommended for those where the model yields negative results. Thirdly, there is a current lack of sufficient information to fully explain the practical utility of GBM models in clinical settings. To address this, future efforts will focus on the enhancement of learning and developing software that is more practical and accurate. The goal is to empower clinicians to easily use these tools online to predict the risk of SIC in patients proactively, facilitating early preventive measures.

## 6 Conclusions

In conclusion, we developed an operable ML prediction model incorporating 17 clinical features to effectively predict the risk of SIC in ICU patients with sepsis. In addition, the prediction model showed good predictive ability and discrimination in the external validation. Nevertheless, further prospective studies are warranted to validate the effectiveness and applicability of this prediction model.

## Data Availability

The data analyzed in this study is subject to the following licenses/restrictions: Hospital datasets generated and analyzed in this study will be available by the corresponding author upon reasonable request. The MIMIC database is a publicly available database. Requests to access these datasets should be directed to GS, srgl985@126.com. https://mimic-iv.mit.edu/.
