# Supplementary material for: A machine learning model for robust prediction of sepsis-induced coagulopathy in critically ill patients with sepsis
Source: Front Cell Infect Microbiol. 2025 Jun 6;15:1579558. doi: 10.3389/fcimb.2025.1579558 (PMC12179180; doi:10.3389/fcimb.2025.1579558)
Supplement: Supplementary file 1 [file Table1.docx]

Supplementary Material

## Supplementary Figures

**Supplementary Table 1**. Sepsis‐induced coagulopathy (SIC) criteria

|  | Score | Range |
| --- | --- | --- |
| Platelet count (−10^9^/L) | 2 | < 100 |
|  | 1 | ≧ 100, < 150 |
| INR | 2 | > 1.4 |
|  | 1 | > 1.2, ≦1.4 |
| SOFA score | 2 | ≧ 2 |
|  | 1 | 1 |
| Total score for SIC |  | ≥4 |

SOFA score is the sum of 4 items (respiratory SOFA, cardiovascular SOFA, hepatic SOFA, renal SOFA). Abbreviations: SIC sepsis‐induced coagulopathy, INR international normalized ratio, SOFA sequential organ failure assessment.

**Supplementary Table 2** Baseline characteristics between the SIC and non-SIC groups in the MIMIC-IV cohort

| **MIMIC-IV set (n=15479)** | | | | |
| --- | --- | --- | --- | --- |
|  | **level** | **non-SIC(9443)** | **SIC(6036)** | **p** |
| **Demographic variables** |  |  |  |  |
| gender (%) | Female | 4239 (44.89) | 2395 (39.68) | <0.001 |
|  | Male | 5204 (55.11) | 3641 (60.32) |  |
| age,median[Q1,Q3] |  | 68.000 [57.000, 79.000] | 71.000 [59.000,81.000] | <0.001 |
| **Comorbidities,n(%)** |  |  |  |  |
| hypertension (%) | No | 5740 (60.79) | 3600 (59.64) | 0.161 |
|  | Yes | 3703 (39.21) | 2436 (40.36) |  |
| diabetes (%) | No | 7690 (81.44) | 4832 (80.05) | 0.035 |
|  | Yes | 1753 (18.56) | 1204 (19.95) |  |
| copd (%) | No | 8535 (90.38) | 5481 (90.81) | 0.399 |
|  | Yes | 908 (9.62) | 555 (9.19) |  |
| Cerebrovascular_disease (%) | No | 9374 (99.27) | 6017 (99.69) | 0.001 |
|  | Yes | 69 (0.73) | 19 (0.31) |  |
| Coronary (%) | No | 7132 (75.53) | 4342 (71.94) | <0.001 |
|  | Yes | 2311 (24.47) | 1694 (28.06) |  |
| liver (%) | No | 9385 (99.39) | 5994 (99.30) | 0.606 |
|  | Yes | 58 (0.61) | 42 (0.70) |  |
| shock (%) | No | 8858 (93.80) | 5306 (87.91) | <0.001 |
|  | Yes | 585 (6.20) | 730 (12.09) |  |
| **Support (1st 24h),n (%)** |  |  |  |  |
| crrt (%) | No | 9206 (97.49) | 5567 (92.23) | <0.001 |
|  | Yes | 237 (2.51) | 469 (7.77) |  |
| heparin (%) | No | 1583 (16.76) | 1490 (24.69) | <0.001 |
|  | Yes | 7860 (83.24) | 4546 (75.31) |  |
| Vasoactive_drug (%) | No | 8710 (92.24) | 5001 (82.85) | <0.001 |
|  | Yes | 733 (7.76) | 1035 (17.15) |  |
| surgery (%) | No | 9429 (99.85) | 6022 (99.77) | 0.317 |
|  | Yes | 14 (0.15) | 14 (0.23) |  |
| mechanical_ventilation (%) | No | 9371 (99.24) | 5983 (99.12) | 0.489 |
|  | Yes | 72 (0.76) | 53 (0.88) |  |
| **Infection Status,n(%)** |  |  |  |  |
| urinary_infection (%) | No | 8117 (85.96) | 5117 (84.77) | 0.044 |
|  | Yes | 1326 (14.04) | 919 (15.23) |  |
| blood_infection (%) | No | 9402 (99.57) | 5986 (99.17) | 0.003 |
|  | Yes | 41 (0.43) | 50 (0.83) |  |
| pulmonary_infection (%) | No | 9423 (99.79) | 6022 (99.77) | 0.932 |
|  | Yes | 20 (0.21) | 14 (0.23) |  |
| **Severity of illness** |  |  |  |  |
| sofa_24hours,median[Q1,Q3] |  | 4.000 [2.000, 6.000] | 4.000 [2.000, 6.000] | 0.182 |
| charlson_comorbidity_index,median[Q1,Q3] |  | 5.000 [3.000, 7.000] | 6.000 [4.000, 8.000] | <0.001 |
| gcs,median[Q1,Q3] |  | 15.000 [15.000, 15.000] | 15.000 [15.000,15.000] | <0.001 |
| **Laboratory tests** |  |  |  |  |
| PLT,median[Q1,Q3] |  | 202.000 [152.000, 269.000] | 151.000 [87.000, 243.000] | <0.001 |
| inr,median[Q1,Q3] |  | 1.100 [1.100, 1.200] | 1.600 [1.400, 2.100] | <0.001 |
| pt,median[Q1,Q3] |  | 12.600 [11.800, 13.550] | 17.250 [15.200,22.600] | <0.001 |
| ptt,median[Q1,Q3] |  | 28.800 [26.300, 32.600] | 34.400 [29.700,44.200] | <0.001 |
| hematocrit，median[Q1,Q3] |  | 35.300 [31.200, 39.600] | 33.400 [29.400,38.000] | <0.001 |
| albumin，median[Q1,Q3] |  | 3.500 [3.000, 3.900] | 3.200 [2.700, 3.600] | <0.001 |
| aniongap，median[Q1,Q3] |  | 15.000 [13.000, 18.000] | 16.000 [14.000,20.000] | <0.001 |
| bicarbonate，median[Q1,Q3] |  | 24.000 [22.000, 27.000] | 24.000 [21.000,26.000] | <0.001 |
| calcium,median[Q1,Q3]) |  | 8.600 [8.100, 9.100] | 8.500 [8.000, 9.000] | <0.001 |
| chloride,median[Q1,Q3]) |  | 106.000 [103.000, 110.000] | 106.000 [101.000,  110.000] | 0.013 |
| creatinine,median[Q1,Q3]) |  | 1.000 [0.800, 1.500] | 1.300 [0.900, 2.100] | <0.001 |
| glucose,median[Q1,Q3]) |  | 141.000 [116.000, 182.000] | 144.000 [116.000, 194.000] | <0.001 |
| sodium,mean(SD) |  | 140.166 (4.831) | 139.778 (5.440) | <0.001 |
| potassium,median[Q1,Q3]) |  | 4.400 [4.000, 4.900] | 4.500 [4.100, 5.000] | <0.001 |
| alt,median[Q1,Q3] |  | 25.000 [15.000, 52.000] | 31.000 [17.000,80.000] | <0.001 |
| alp,median[Q1,Q3] |  | 78.000 [59.000, 107.000] | 91.000 [65.000,  141.000] | <0.001 |
| ast,median[Q1,Q3] |  | 35.000 [22.000, 71.000] | 49.000 [27.000, 131.000] | <0.001 |
| bilirubin_total,median[Q1,Q3] |  | 0.600 [0.400, 1.000] | 0.900 [0.500, 1.800] | <0.001 |
| hemoglobin,mean(SD) |  | 11.220 (2.217) | 10.618 (2.305) | <0.001 |
| mch,median[Q1,Q3] |  | 30.200 [28.700, 31.600] | 30.200 [28.500,31.700] | 0.240 |
| mchc,mean(SD) |  | 32.948 (1.653) | 32.639 (1.743) | <0.001 |
| mcv,mean(SD) |  | 91.061 (6.724) | 92.097 (7.737) | <0.001 |
| rbc,mean(SD) |  | 3.759 (0.746) | 3.555 (0.795) | <0.001 |
| rdw,median[Q1,Q3] |  | 14.100 [13.200, 15.400] | 15.000 [13.800,17.000] | <0.001 |
| wbc,mean(SD) |  | 11.541 (6.516) | 12.524 (14.681) | <0.001 |
| **Vital signs** |  |  |  |  |
| heart_rate,mean(SD) |  | 83.385 (15.288) | 87.266 (16.889) | <0.001 |
| sbp,median[Q1,Q3] |  | 117.920 [108.195, 129.895] | 111.045 [103.258, 121.305] | <0.001 |
| dbp,median[Q1,Q3] |  | 61.890 [55.785, 69.220] | 59.965 [54.020,67.120] | <0.001 |
| mbp,median[Q1,Q3] |  | 77.630 [71.745, 85.150] | 74.480 [69.040,81.112] | <0.001 |
| resp_rate,median[Q1,Q3] |  | 18.460 [16.520, 20.910] | 19.320 [17.040,22.360] | <0.001 |
| temperature,median[Q1,Q3] |  | 36.850 [36.630, 37.130] | 36.790 [36.550,37.090] | <0.001 |
| spo2,median[Q1,Q3] |  | 97.190 [95.850, 98.480] | 97.130 [95.667,98.420] | <0.001 |

Abbreviations: SIC sepsis-induced coagulopathy, MIMIC-Ⅳ Medical Information Mart for Intensive Care Ⅳ， SD standard deviation, COPD Chronic Obstructive Pulmonary Disease, SOFA sequential organ failure assessment, GCS Glasgow Coma Scale, PLT Platelet Count, PTT Partial Thromboplastin Time, ALT Alanine Aminotransferase, MBP Mean Blood Pressure, BUN blood urea nitrogen, INR international normalized ratio, PT prothrombin time, RDW red cell distribution width, WBC white blood cell count, SpO_2_ Peripheral capillary oxygen saturation, CRRT continuous renal replacement therapy, MCV Mean Corpuscular Volume, ALP Alkaline Phosphatase, AST Aspartate Aminotransferase, MCH Mean Corpuscular Hemoglobin, MCHC Mean Corpuscular Hemoglobin Concentration, RBC Red Blood Cell, SBP Systolic Blood Pressure, DBP Diastolic Blood Pressure, Resp_rate Respiratory Rate

**Supplementary Table 3** Results of Logistic Regression

| name | desc | Non-SIC (N=6611) | SIC (N=4226) | OR (95%CI, P) [univariable] | OR (95%CI, P)  [multivariable] |
| --- | --- | --- | --- | --- | --- |
| gender | Female | 2983 (45.1%) | 1678 (39.7%) |  |  |
|  | Male | 3628 (54.9%) | 2548 (60.3%) | 1.25 (1.15-1.35, p<.001) | 1.09 (0.96-1.25, p=.189) |
| hypertension | No | 3999 (60.5%) | 2527 (59.8%) |  |  |
|  | Yes | 2612 (39.5%) | 1699 (40.2%) | 1.03 (0.95-1.11, p=.472) |  |
| diabetes | No | 5379 (81.4%) | 3380 (80%) |  |  |
|  | Yes | 1232 (18.6%) | 846 (20%) | 1.09 (0.99-1.20, p=.074) |  |
| copd | No | 5976 (90.4%) | 3857 (91.3%) |  |  |
|  | Yes | 635 (9.6%) | 369 (8.7%) | 0.90 (0.79-1.03, p=.126) |  |
| Cerebrovascular_disease | No | 6558 (99.2%) | 4210 (99.6%) |  |  |
|  | Yes | 53 (0.8%) | 16 (0.4%) | 0.47 (0.27-0.82, p=.008) | 0.81 (0.34-1.92, p=.625) |
| Coronary | No | 4958 (75%) | 3034 (71.8%) |  |  |
|  | Yes | 1653 (25%) | 1192 (28.2%) | 1.18 (1.08-1.29, p<.001) | 0.78 (0.67-0.91, p=.002) |
| liver | No | 6564 (99.3%) | 4199 (99.4%) |  |  |
|  | Yes | 47 (0.7%) | 27 (0.6%) | 0.90 (0.56-1.44, p=.657) |  |
| crrt | No | 6433 (97.3%) | 3875 (91.7%) |  |  |
|  | Yes | 178 (2.7%) | 351 (8.3%) | 3.27 (2.72-3.94, p<.001) | 1.53 (1.10-2.12, p=.012) |
| heparin | No | 1072 (16.2%) | 1016 (24%) |  |  |
|  | Yes | 5539 (83.8%) | 3210 (76%) | 0.61 (0.56-0.67, p<.001) | 0.54 (0.46-0.64, p<.001) |
| Vasoactive_drug | No | 6099 (92.3%) | 3483 (82.4%) |  |  |
|  | Yes | 512 (7.7%) | 743 (17.6%) | 2.54 (2.25-2.87, p<.001) | 1.44 (1.17-1.77, p<.001) |
| shock | No | 6183 (93.5%) | 3691 (87.3%) |  |  |
|  | Yes | 428 (6.5%) | 535 (12.7%) | 2.09 (1.83-2.39, p<.001) | 0.84 (0.67-1.06, p=.141) |
| surgery | No | 6598 (99.8%) | 4219 (99.8%) |  |  |
|  | Yes | 13 (0.2%) | 7 (0.2%) | 0.84 (0.34-2.11, p=.714) |  |
| mechanical_ventilation | No | 6556 (99.2%) | 4188 (99.1%) |  |  |
|  | Yes | 55 (0.8%) | 38 (0.9%) | 1.08 (0.71-1.64, p=.711) |  |
| urinary_infection | No | 5683 (86%) | 3575 (84.6%) |  |  |
|  | Yes | 928 (14%) | 651 (15.4%) | 1.12 (1.00-1.24, p=.049) | 1.06 (0.88-1.27, p=.561) |
| blood_infection | No | 6579 (99.5%) | 4198 (99.3%) |  |  |
|  | Yes | 32 (0.5%) | 28 (0.7%) | 1.37 (0.82-2.28, p=.224) |  |
| pulmonary_infection | No | 6596 (99.8%) | 4214 (99.7%) |  |  |
|  | Yes | 15 (0.2%) | 12 (0.3%) | 1.25 (0.59-2.68, p=.562) |  |
| age | Mean ± SD | 66.8 ± 16.7 | 68.7 ± 15.7 | 1.01 (1.00-1.01, p<.001) | 1.00 (1.00-1.01, p=.787) |
| charlson_comorbidity_index | Mean ± SD | 5.0 ± 3.0 | 5.8 ± 2.9 | 1.09 (1.07-1.10, p<.001) | 1.00 (0.97-1.02, p=.767) |
| gcs | Mean ± SD | 14.2 ± 2.1 | 14.2 ± 2.3 | 1.00 (0.98-1.02, p=.894) |  |
| pt | Mean ± SD | 12.6 ± 1.5 | 20.6 ± 10.2 | 2.81 (2.69-2.93, p<.001) | 2.78 (2.65-2.91, p<.001) |
| ptt | Mean ± SD | 33.2 ± 15.5 | 42.1 ± 22.0 | 1.03 (1.03-1.03, p<.001) | 1.00 (1.00-1.00, p=.856) |
| hematocrit | Mean ± SD | 35.7 ± 6.0 | 34.0 ± 6.3 | 0.96 (0.95-0.96, p<.001) | 0.99 (0.97-1.00, p=.108) |
| albumin | Mean ± SD | 3.4 ± 0.6 | 3.2 ± 0.7 | 0.55 (0.52-0.58, p<.001) | 0.98 (0.88-1.09, p=.708) |
| aniongap | Mean ± SD | 16.3 ± 4.7 | 17.5 ± 5.8 | 1.05 (1.04-1.05, p<.001) | 1.02 (1.01-1.04, p=.013) |
| bicarbonate | Mean ± SD | 24.7 ± 4.1 | 24.0 ± 4.6 | 0.96 (0.95-0.97, p<.001) | 0.99 (0.97-1.01, p=.353) |
| bun | Mean ± SD | 27.2 ± 21.9 | 35.3 ± 26.6 | 1.01 (1.01-1.02, p<.001) | 1.00 (1.00-1.01, p=.100) |
| calcium | Mean ± SD | 8.6 ± 0.7 | 8.6 ± 1.4 | 0.94 (0.89-0.98, p=.008) | 0.96 (0.88-1.05, p=.369) |
| chloride | Mean ± SD | 106.0 ± 6.2 | 105.6 ± 7.0 | 0.99 (0.98-1.00, p=.002) | 1.02 (1.00-1.04, p=.096) |
| creatinine | Mean ± SD | 1.6 ± 1.9 | 1.9 ± 1.7 | 1.10 (1.07-1.13, p<.001) | 0.96 (0.91-1.01, p=.130) |
| glucose | Mean ± SD | 169.7 ± 105.5 | 174.3 ± 103.9 | 1.00 (1.00-1.00, p=.026) | 1.00 (1.00-1.00, p=.468) |
| sodium | Mean ± SD | 140.2 ± 4.8 | 139.7 ± 5.4 | 0.98 (0.97-0.99, p<.001) | 0.98 (0.96-1.00, p=.091) |
| potassium | Mean ± SD | 4.6 ± 0.8 | 4.7 ± 0.9 | 1.19 (1.14-1.24, p<.001) | 0.98 (0.90-1.07, p=.676) |
| alt | Mean ± SD | 118.6 ± 592.5 | 255.1 ± 1008.0 | 1.00 (1.00-1.00, p<.001) | 1.00 (1.00-1.00, p=.799) |
| alp | Mean ± SD | 104.3 ± 111.6 | 131.2 ± 154.7 | 1.00 (1.00-1.00, p<.001) | 1.00 (1.00-1.00, p=.637) |
| ast | Mean ± SD | 162.5 ± 825.3 | 451.3 ± 1866.8 | 1.00 (1.00-1.00, p<.001) | 1.00 (1.00-1.00, p=.091) |
| bilirubin_total | Mean ± SD | 1.0 ± 1.9 | 2.1 ± 3.9 | 1.24 (1.21-1.27, p<.001) | 1.07 (1.04-1.11, p<.001) |
| hemoglobin | Mean ± SD | 11.2 ± 2.2 | 10.6 ± 2.3 | 0.89 (0.87-0.90, p<.001) | 1.11 (0.92-1.33, p=.273) |
| mch | Mean ± SD | 30.0 ± 2.6 | 30.1 ± 2.9 | 1.01 (1.00-1.03, p=.055) |  |
| mchc | Mean ± SD | 32.9 ± 1.7 | 32.6 ± 1.7 | 0.90 (0.88-0.92, p<.001) | 0.97 (0.90-1.04, p=.413) |
| mcv | Mean ± SD | 91.0 ± 6.7 | 92.2 ± 7.8 | 1.02 (1.02-1.03, p<.001) | 1.02 (1.00-1.05, p=.050) |
| rbc | Mean ± SD | 3.8 ± 0.7 | 3.6 ± 0.8 | 0.70 (0.67-0.74, p<.001) | 0.67 (0.39-1.14, p=.142) |
| rdw | Mean ± SD | 14.6 ± 1.9 | 15.6 ± 2.5 | 1.25 (1.23-1.27, p<.001) | 1.13 (1.09-1.17, p<.001) |
| wbc | Mean ± SD | 11.5 ± 6.7 | 12.5 ± 14.8 | 1.01 (1.00-1.01, p<.001) | 1.00 (1.00-1.01, p=.565) |
| heart_rate | Mean ± SD | 83.4 ± 15.2 | 87.3 ± 16.9 | 1.02 (1.01-1.02, p<.001) | 1.00 (1.00-1.01, p=.758) |
| sbp | Mean ± SD | 120.0 ± 16.0 | 113.4 ± 15.2 | 0.97 (0.97-0.98, p<.001) | 0.98 (0.98-0.99, p<.001) |
| dbp | Mean ± SD | 63.1 ± 10.8 | 61.1 ± 10.4 | 0.98 (0.98-0.99, p<.001) | 0.99 (0.97-1.00, p=.132) |
| mbp | Mean ± SD | 78.9 ± 10.7 | 75.7 ± 10.1 | 0.97 (0.97-0.97, p<.001) | 1.04 (1.01-1.06, p=.002) |
| resp_rate | Mean ± SD | 19.1 ± 3.6 | 20.0 ± 4.1 | 1.07 (1.06-1.08, p<.001) | 1.02 (1.00-1.04, p=.029) |
| temperature | Mean ± SD | 36.9 ± 0.5 | 36.8 ± 0.6 | 0.79 (0.74-0.85, p<.001) | 1.12 (1.00-1.26, p=.053) |
| spo2 | Mean ± SD | 97.0 ± 2.0 | 96.8 ± 2.4 | 0.96 (0.94-0.98, p<.001) | 1.01 (0.98-1.04, p=.512) |

In the multivariable logistic regression analysis, variables with statistical significance (p < 0.05) are highlighted in red. Prothrombin time (PT) was excluded from the final model, whereas blood urea nitrogen (BUN), diabetes mellitus, serum sodium, aspartate aminotransferase (AST), mean corpuscular hemoglobin (MCH), and body temperature were retained in the model and marked in blue. Abbreviations: SIC sepsis-induced coagulopathy, OR Odds Ratio, 95%CI 95% Confidence Interval, COPD Chronic Obstructive Pulmonary Disease, GCS Glasgow Coma Scale, PTT Partial Thromboplastin Time, ALT Alanine Aminotransferase, MBP Mean Blood Pressure, BUN blood urea nitrogen, PT prothrombin time, RDW red cell distribution width, WBC white blood cell count, SpO_2_ Peripheral capillary oxygen saturation, CRRT continuous renal replacement therapy, MCV Mean Corpuscular Volume, ALP Alkaline Phosphatase, AST Aspartate Aminotransferase, MCH Mean Corpuscular Hemoglobin, MCHC Mean Corpuscular Hemoglobin Concentration, RBC Red Blood Cell, SBP Systolic Blood Pressure, DBP Diastolic Blood Pressure, Resp_rate Respiratory Rate
